# Influence of mental health on information seeking, risk perception and mask wearing self-efficacy during the early months of the COVID-19 pandemic: a longitudinal panel study across 6 U.S. States

**DOI:** 10.1186/s40359-023-01241-z

**Published:** 2023-07-10

**Authors:** Courtney Welton-Mitchell, Miranda Dally, Katherine L. Dickinson, Lindsay Morris-Neuberger, Jennifer D. Roberts, Danielle Blanch-Hartigan

**Affiliations:** 1 Colorado School of Public Health, University of Colorado, Mail Stop, 13001 E 17th Pl B119, Aurora, CO 80045 USA; 2 Communication Studies, West Virginia University, Armstrong Hall, 94 Beechurst Ave STE 108, Morgantown, WV 26505 USA; 3 School of Public Health, University of Maryland, 4200 Valley Dr, College Park, MD 20742 USA; 4 Natural and Applied Sciences, Bentley University, 175 Forest St, Waltham, MA 02452 USA

**Keywords:** COVID-19, Mental health, Information seeking, Risk perception, Mask wearing

## Abstract

**Background:**

Understanding factors that influence information seeking, assessment of risk and mitigation behaviors is critical during a public health crises. This longitudinal study examined the influence of self-reported mental health during the early months of the COVID-19 pandemic on information seeking, risk perception and perceived mask wearing ability. Mental health screener items included fear, anger, and hopelessness in addition to avoidance, diminished functional ability and global distress. Theoretical models inform hypotheses linking mental health items and outcomes.

**Methods:**

The research employed a longitudinal 6-state 3-wave online panel survey, with an initial sample of 3,059 participants (2,232 included in longitudinal analyses). Participants roughly represented the states’ age, race, ethnicity, and income demographics.

**Results:**

Women, those who identified as Hispanic/Latinx, Black Americans and lower income participants reported higher overall rates of distress than others. Information seeking was more common among older persons, Democrats, retirees, those with higher education, and those who knew people who had died of COVID-19. Controlling for such demographic variables, in multivariable longitudinal models that included baseline mental health measures, distress and fear were associated with increased information seeking. Distress and fear were also associated with increased risk perception, and feelings of hopelessness were associated with lower reported mask-wearing ability.

**Conclusions:**

Results advance understanding of the role mental health can play in information seeking, risk perception and mask wearing with implications for clinicians, public health practitioners and policy makers.

**Supplementary Information:**

The online version contains supplementary material available at 10.1186/s40359-023-01241-z.

## Background

With an increase in anxiety and depression symptomatology, mental health appears to have worsened globally and in the U.S. among many groups during the first few years of the pandemic [1–6]. Research indicates that death of a loved one, job loss and increased isolation may be some of the drivers of pandemic-related distress [7–9]. Women, youth, Black Americans, Hispanic/Latinx and other historically marginalized groups appear to have fared worse than others [1, 10–13]. Prevalence estimates are, however, a matter of debate, due in part to methodological differences across studies [14, 15].

The COVID-19 pandemic intersects with a continuing global mental health crisis in complex and multidirectional ways. Psychological factors are intertwined with individual responses to COVID-19. The present study examines the linkages between mental health and these responses, specifically information seeking, risk perception, and self-efficacy associated with risk mitigation behavior.

### Mental health and information seeking

Health information seeking, ideally from reputable, credible sources, is an important step in influencing health perceptions and behaviors [16]. Research indicates that health information seeking is associated with discussion of information with health care providers and knowledge about treatment options, although it may also pose a risk of self-diagnosis and non-adherence [17]. Psychological factors can enhance or undermine health related information seeking [18]. Mental health symptoms can influence whether people seek out or avoid information, and how they perceive risk and mitigation behaviors [19–24]. Psychological distress has been associated with health information seeking, even after controlling for health status [24]. Seeking health-related information is a common response among those facing health threats [25, 26]. In a meta-analysis involving 20 studies and over 7,000 participants, there was a consistent positive relationship between health anxiety and health-related information seeking [27]. Anxiety and fear, including fear of illness, can motivate information seeking, which may even serve as a strategy for decreasing anxiety, especially in situations with high ambiguity [22, 23, 28, 29].

However, some individuals may respond to health information with heightened anxiety, especially if they believe they are unable to control the situation [30]. Avoidant coping, including avoidance of health-related information, may be used in an attempt to mitigate this anxiety, especially among those with previous experience of serious illness [31–33]. Depression has also been associated with avoidance coping as a strategy to mitigate emotional distress [34–37]. Additionally, anger, correlated with both depression and anxiety [38], has been linked to information avoidance [39].

### Mental health and health risk perception

Evidence suggests that an individual’s perceived susceptibility to a threat is often a driver of health behaviors designed to mitigate risk [40]. Mental health may influence perception of health risks, such as those posed by COVID-19. Factors associated with health-related risk perception include gender, age, health status, socioeconomic status, education, disease knowledge, exposure to disease related information, disease experience, and trust in relevant stakeholders providing information [41]. Psychological distress has also been associated with increased health risk perception [20, 42], including in a scoping review of perceived risk of respiratory infectious diseases [41]. Depressive symptoms specifically have been linked to greater perception of health risks, and more pessimistic health appraisals overall [43]. Affective states such as fear have also been associated with more pessimistic risk perceptions, whereas those experiencing anger may have more optimistic appraisals, resulting in lower perception of risk [19]. In a study of psychological factors and risk perception among Italians during the COVID-19 pandemic, however, negative affective states including fear, sadness, anxiety and anger were all associated with increased perception of risk [44].

### Mental health and risk mitigation behavior

Risk mitigation measures are important in limiting the spread of disease [45]. Risk mitigation and inversely, risky behaviors, have been associated with factors including mental health, affective states, attitudes, beliefs and perceived control [44, 46–48]. These research findings extend to health risk mitigation and risk mitigation in emergency situations, both of which are relevant to the COVID-19 pandemic. Fear and worry have been linked to increased compliance with guidance during the COVID-19 pandemic [47, 49–51]. Psychological distress and depression, however, have been linked to decreased likelihood of engaging in routine health risk mitigation, such as cancer screenings [21, 52], although this relationship may be moderated by age [53]. Anxiety and avoidant coping have also been associated with a lower likelihood of engaging in risk mitigation behaviors associated with emergencies, such as in disaster preparedness [54, 55].

Psychological factors, such as self-efficacy, appear to be a necessary precursor for some risk mitigation behaviors. Confidence in one’s ability to overcome barriers and take action to mitigate risk can influence the decision to act [56]. During the COVID-19 pandemic, mask wearing compliance has been linked to a belief that health precautions can be undertaken and will be effective [57–59]. Risk mitigation-related self-efficacy has also been associated with better mental health during the pandemic [60, 61].

### The role of affect

Theoretical frameworks associated with information seeking, risk perception, and risk mitigation beliefs and behaviors typically emphasize the importance of affective states. Affective states such as fear, anger, and sadness, are among the defining features of many mental health conditions [62] and are typically a focus of questions on mental health screeners. As such, theoretical models that underscore the importance of affect can illuminate potential relationships between mental health and other variables of interest.

The *General Model of Societal Risk Information Seeking and Avoiding* [63], suggests information seeking is a result of several factors, including individual characteristics (e.g., demographics, experience, values), cognitive, affective and situational precursors. This model adapts frameworks focused on information-seeking specific to individual risks, to incorporate broader ‘societal risks’ (e.g., climate change, pandemics). The model also includes testable propositions, such as the hypothesis that affective responses towards the hazard will influence information seeking.

The *Conceptual Model of Health Risk Perceptions* [64] suggests that discrete emotions such as fear, anger, and sadness influence risk perception. Similarly, the *Appraisal Tendency Framework (ATF)* emphasizes the motivational properties associated with discrete emotions, such as fear or worry, that can influence judgment and decision-making [65]. In contrast, the *Valence Theory of Risk Perception* [66] indicates that what matters when considering risk is not the specific emotion, but whether the emotion is negative or positive.

### Current study

Understanding drivers of information-seeking, risk perception and risk mitigation behaviors during the early months of a disease outbreak is an urgent public health priority. To date, however, there is a gap in research examining the role mental health symptoms play in how people engage with, interpret and act on information about public health threats over time. This longitudinal panel research addresses this gap, focusing on the early months of the COVID-19 pandemic.

Hypotheses related to mental health and information seeking were informed by the *General Model of Societal Risk Information Seeking and Avoiding* [63], with the direction of anticipated effects consistent with previous research. Specifically:

- H1: distress and fear symptoms will be associated with increased information seeking.
- H2: avoidance symptoms will be associated with decreased information seeking.

Hypotheses related to mental health and risk perception, and risk mitigation self-efficacy, are consistent with the *Conceptual Model of Health Risk Perceptions* [64] and, at a more granular level, the *Valence Theory of Risk Perception* [66]. Alternative hypotheses, are consistent with the *Appraisal Tendency Framework* [65]. Specifically:

- H3: negative emotions (distress, fear, anger and depressive symptoms - disinterest in usual activities and hopelessness) will be associated with greater risk perception.
  - H3 (alternative): The relationship between specific affective states and risk perception will vary across items.
- H4: negative emotions (distress, fear, anger and depressive symptoms - disinterest in usual activities and hopelessness) will be associated with decreased self-efficacy related to mask wearing.
  - H4 (alternative): Specific affective states will be associated with self-efficacy related to mask wearing, with differences in the direction of association by item.

## Methods

### Sample and procedure

The research was conducted as part of a longitudinal 6-state 3-wave online panel survey with an initial sample of 3,059 participants. Using GPower 3.1, we conducted a power analysis based on estimated indicator variables and a multi-level modeling analytical approach, revealing a need for ~ 500 respondents per state. Data was collected from respondents in: Colorado, Iowa, Louisiana, Massachusetts, Michigan, and Washington. States were selected that were emerging COVID-19 hotspots at the time of survey development, and demonstrated variation in the timing and severity of the COVID-19 pandemic onset, risk reduction policies (including stay-at-home orders and mask mandates), and demographic, political, and social factors.

The online survey panels utilized a proprietary prearranged pool of respondents recruited by QualtricsXM. Survey panel participants were selected based on census categories and were roughly representative of the states’ age, race, ethnicity, and income demographics. However, the sample includes a slightly higher percentage of women due to the difficulty of meeting multiple sampling quotas simultaneously (see ‘Results’ for sample details).

We report on Wave 1, 2 and 3 data, collected in the early months of the pandemic, May-October 2020. The first wave of the survey was conducted between May 15 and June 7, 2020 (N = 3,059). The second wave of the survey was conducted between August 6 and August 25, 2020 (N = 2,078). The third wave was fielded between October 9 and October 21, 2020 (N = 1,794). The same respondents were engaged over each wave. While differential attrition by subgroups did occur during Waves 2 and 3, this is addressed in the supplemental materials (Annex I), comparing those included in the longitudinal cohort and those lost to follow up after the first survey.

### Measures

This study is part of a larger online panel survey, which sought to measure changes in respondents’ COVID-19 perceptions and behaviors over time during the early months of the pandemic. Survey respondents were asked to answer a broad range of questions, including questions about COVID-19-related policies, physical and mental health, information seeking, risk mitigation beliefs and behaviors. For the purposes of this manuscript, we examined associations between mental health and information-seeking, risk perception and mask wearing ability (self-efficacy). Many of the variables utilized in this manuscript were collected in every Wave (1, 2, 3). For theoretical reasons, however, only baseline mental health and demographic variables are utilized in final models, whereas for outcome measures data from all waves is included to examine relationships with baseline variables across time.

#### Demographic information

The survey included information on gender, age, education, income, work status, political affiliation, race and ethnicity. Data on race and ethnicity was collected by asking ‘What is your race?’ and ‘Are you Hispanic or Latino/a/x?’ Race options (select all that apply) were White, Black American, American Indian / Alaskan Native, Asian, Native Hawaiian / Pacific Islander, Other. Racial and ethnic categories were combined during analysis into five distinct categories: Hispanic/Latinx, Non-Hispanic/Latinx Asian, Non-Hispanic/Latinx Black American, Non-Hispanic/Latinx Other or Multiracial, Non-Hispanic/Latinx White. Additionally, information was collected on participant experience with COVID-19, including knowing someone who died from COVID- 19.

#### Mental health indicators - distress thermometer

Distress thermometers have been used for clinical practice and research and can provide a rapid global assessment of mental health [67]. Participants were asked, *Please describe how much distress you have been feeling overall in the past week, where 0 = things are good, 10 = I feel as bad as I have ever felt.*

#### Mental health indicators - WASSS-6 [68]

The WHO-UNHCR Assessment Schedule of Serious Symptoms in Humanitarian Settings (WASSS) was created as a rapid screener for use during disasters. It includes five items related to mental health symptoms (fear, anger, lack of interest, hopelessness, avoidance) and one item measuring functional ability associated with activities essential for daily living. Participants were asked, *In general over the past two weeks, how often have you felt… So afraid that nothing could calm me down; So angry that I felt out of control; Uninterested in things that I used to like; So hopeless that I did not want to carry on living; So upset (about COVID-19) that I tried to avoid conversations or activities that reminded me (of COVID-19); Unable to carry out essential activities for daily living because of the above.* Respondents were asked to rate how frequently they experienced each item during the prior two weeks using the following scale: (1) none of the time; (2) a little of the time; (3) some of the time; (4) most of the time; and (5) all of the time. The avoidance item was adapted to be specific to COVID-19.

#### Information seeking

Participants were asked, *In the past two months how closely have you been following news and information about COVID-19?* Response options ranged from 1 (not at all) to 5 (very closely).

#### Risk perception

Participants were asked, *On a scale of 0-100%, what do you think is the chance that you will get COVID-19 in the next three months? Please give your best guess.* Participants could move a slider from 0-100%. In this dataset, the mode was 50%, the median value was 21% with an interquartile range (IQR) of 40%.

#### Risk mitigation-related self-efficacy

Self-efficacy provides a window into upstream psychological processes and appears to be a necessary precursor for risk mitigation behaviors. Participants were asked, *To what extent do you agree or disagree with the following statement? I am able to wear a face covering.* Response options ranged from 1 (strongly disagree) to 5 (strongly agree). Although respondents were also asked how frequently they wore a mask in indoor public spaces this variable was not used for analyses because we only had data from a non representative subsample of those who were actually visiting indoor public spaces [69]. In contrast, the full sample could respond to a question on mask wearing ability, including those who may have been entirely or mostly avoiding indoor spaces, especially during the initial months of the pandemic.

### Analysis

Statistical analyses were conducted using SAS software, version 9.4, SAS System for Windows (copyright© 2013 SAS Institute Inc.). Baseline differences between those lost to follow-up after Wave 1 and those participating in the cohort at multiple time points were determined using t-tests for continuous variables and Chi-squared tests for proportions. Associations between demographic variables and information seeking at baseline were assessed using linear mixed models with random intercept for individual. Demographic variables deemed to be statistically significant at the 0.001 level were included as adjustment variables in all models to assess the a priori hypotheses.

Effects of mental health variables at baseline on information seeking (H1, H2), risk perception (H3), and risk mitigation self-efficacy (H4) across time were analyzed using linear mixed models. A univariable model for the association between each mental health variable on each hypothesized outcome was constructed (22 total models). The models were constructed with the mental health variables at baseline treated as continuous variables, time (three levels: baseline as reference, Time 2, Time 3) and the interaction of mental health variable and time as fixed factors, and were adjusted for significant demographics. Random intercepts for individual were included in all models to account for correlations between repeated measurements. Final multivariable models were assessed for each outcome. Mental health at baseline variables deemed statistically significant in the univariable model for each outcome were included. For each model, estimates (B) were determined with 95% confidence intervals (CI). To adjust for multiple comparisons the level of statistical significance for the interaction effect was set at a conservative 0.001.

## Results

### Sample characteristics across waves

There were 3,059 unique respondents. Of these, 827 (27%) participated in only the baseline survey and were excluded from the analysis. The remaining 2,232 survey respondents included in longitudinal analyses were 63% women with a mean age of 43.28 (SD = 15.58), and household size of 3.08 (SD = 1.64). The majority of respondents were Non-Hispanic/Non-Latinx (NHNL) White (73%), with 10% NHNL Black Americans, 10% Hispanic/Latinx, 5% NHNL Asian, 4% NHNL Other or Multiracial. Almost half of respondents (49%) had an income under $60,000 per year. Just over two thirds of the sample (79%) had at least ‘some college’. The sample was fairly evenly split by political party, with 36% of respondents identifying as Democrat, 25% Republican, 34% Independent, and 6% Other. Over half (57%) were employed at least part-time, 18% were unemployed or furloughed, 13% were retirees, 5% were students, 4% were working without pay, and 4% were on disability. As a reminder, sampling quotas were used to ensure that the sample demographics were similar to each state’s age, race, ethnicity, and income demographics. Despite this, based on 2021 nationwide census data [70], there is some overrepresentation of women and those with some college or higher education (although income levels are comparable or lower than nationwide data). There is also some underrepresentation of Black Americans and Hispanic/Latinx groups.

The baseline and longitudinal samples were compared to better understand those lost to follow up. On average, those participating in only the baseline survey were angrier [1.70 (SD: 0.97) vs. 1.61 (SD: 0.88)] and had less functional ability [1.90 (SD: 1.15) vs. 1.70 (SD: 0.98)] than those who participated in follow-up surveys. More women participated in the follow-up surveys (63% versus 53%; p-value < 0.001) and Non-Hispanic/Non-Latinx Whites were overrepresented in follow-up surveys (73% versus 65%; p-value < 0.001). For additional details on demographics and all comparisons between those included in the longitudinal cohort and those lost to follow up after baseline, see Supplemental Materials, Annex I.

Table 1 summarizes baseline mental health outcomes and demographics associated with distress. Distress and responses to mental health screener items were relatively high, with 41% of respondents endorsing a 6 or higher on the distress thermometer (0–10) scale, and between 11% and 33% of the sample with a response indicating at least ‘some of the time’ on each of the mental health screener items. Notably, when used in clinical practice, a distress rating of at least 4 and any response of ‘some of the time’ or above is considered a ‘red flag’ in need of follow up [71, 72]. Although these rates appeared fairly constant across the 3 waves, there was differential attrition among specific subgroups. Notably, some groups reported higher rates of distress than others including women, younger participants, Hispanic/Latinx, Black American respondents, lower income groups, and those who know people who have died of COVID-19. Groups both working and unable to work during the pandemic fared worse when compared to retirees. Republicans reported less distress compared to Democrats.

**Table 1:** Baseline Mental health descriptives and demographics associated with distress

Mental health survey items	T1 (May/June 2020) (total sample N = 3,059) (*M*; *SD*)(n)
Distress overall in past week (6 or higher rating out of 10, 0 = things are good, 10 = I feel as bad as I have ever felt)	41% (*M* = 0.41; *SD* = 0.49)(n = 3.056)
*Mental Health Screener items, past 2 weeks, how often have you felt…* *(% reporting ‘some, most, all of the time ’ collapsed)*	
So afraid that nothing could calm me down (fear)	17% (*M* = 0.17; *SD* = 0.38)(n = 3,002)
So angry that I felt out of control (anger)	18% (*M* = 0.18; *SD* = 0.38)(n = 3,009)
Uninterested in things that I used to like (disinterest)	33% (*M* = 0.32; *SD* = 0.47)(n = 3,010)
So hopeless that I did not want to carry on living (hopelessness)	11% (*M* = 0.11; *SD* = 0.32)(n = 3,010)
So upset about COVID-19 that I tried to avoid conversations or activities that reminded me of COVID-19 (upset/avoidance)	28% (*M* = 0.28; *SD* = 0.45)(n = 3,010)
Unable to carry out activities for essential living because of the above (diminished functional ability)	23% (*M* = 0.22; *SD* = 0.42)(n = 3,010)
**Association between demographics and distress at Time 1 (REF = reference group)**	**B (CI), p value**
Gender (Female )	0.82 (0.62, 1.02), < 0.001
Age	-0.04 (-0.05, -0.04), < 0.001
Hispanic/Latinx (REF: White)	0.81 (0.48, 1.13), < 0.001
Asian (REF: White)	0.30 (-0.21, 0.81), 0.251
Black American (REF: White)	0.88 (0.56, 1.20), < 0.001
Multiracial (REF: White)	0.55 (0.03, 1.06), 0.039
Less than high school (REF: Graduate degree)	0.76 (0.11, 1.04), 0.021
High school (REF: Graduate degree)	0.67 (0.35, 1.00), < 0.001
Some college (REF: Graduate degree)	0.29 (-0.02, 0.61), 0.068
2 year degree (REF: Graduate degree)	0.70 (0.32, 1.08), < 0.001
4 year degree (REF: Graduate degree)	0.25 (-0.06, 0.56), 0.118
$10,000 or less (REF: More than $150,000)	1.14 (0.70, 1.59), < 0.001
$10,001-$20,000 (REF: More than $150,000)	1.43 (0.97, 1.89), < 0.001
$20,001-$30,000 (REF: More than $150,000)	0.91 (0.45, 1.38), < 0.001
$30,001 to $40,000 (REF: More than $150,000)	1.00 (0.53, 1.46), < 0.001
$40,001 to $50,000 (REF: More than $150,000)	0.70 (0.24, 1.15), 0.003
$50,001 to $60,000 (REF: More than $150,000)	0.37 (-0.10, 0.85), 0.119
$60,001 to $80,000 (REF: More than $150,000)	0.22 (-0.19, 0.64), 0.293
$80,001 to $100,000 (REF: More than $150,000)	0.34 (-0.08, 0.77), 0.115
$100,001 to $150,000 (REF: More than $150,000)	0.20 (-0.12, 0.64), 0.182
Employed full time (32 or more hours/week) (REF: Retired)	1.30 (1.00, 1.61), < 0.001
Employed part time (1–31 h/week) (REF: Retired)	1.49 (1.11, 1.87), < 0.001
Working without pay (e.g., childcare, volunteering) (REF: Retired)	1.51 (0.94, 2.07), < 0.001
Furloughed (REF: Retired)	1.35 (0.81, 1.89), < 0.001
Unemployed and looking for work (REF: Retired)	2.28 (1.85, 2.72), < 0.001
Unemployed and not looking for work (REF: Retired)	1.40 (0.89, 1.90), < 0.001
Receiving or awaiting approval for disability payments (REF: Retired)	2.58 (2.00, 3.16), < 0.001
Primarily a student (REF: Retired)	2.01 (1.49, 2.54), < 0.001
Republican (REF: Democrat)	-0.88 (-1.14, -0.62), < 0.001
Independent (REF: Democrat)	-0.34 (-0.57, -0.10), 0.005
Other (REF: Democrat)	-0.08 (-0.51, 0.36), 0.730
#Known who have died of COVID-19	0.59 (0.33, 0.89), < 0.001

T1: N = 3,056; T2: N = 2,078; T3: N = 1,794. Variables performed fairly consistently across the 3 time points. However, we only report Time 1 due to differential attrition across time among specific subgroups (men, Non-White respondents). In addition, on average those participating in only T1 were angrier and reported less functional ability than those who participated in follow-up surveys. Relationships were similar between Time 1 demographics and distress across time

Table 2 summarizes demographic associations with information seeking at baseline. Notably, on average older persons sought information more than younger persons (B (CI): 0.01 (0.01, 0.02)), and those who had less education sought information less frequently compared to those with graduate degrees (B (CI): -0.69 (-1.00, -0.38)). Individuals identifying with the Democratic party sought information more frequently than individuals identifying with other political parties (B (CI): 0.24 (0.13, 0.36)). Employment status had a significant effect on information seeking frequency with retirees seeking information most frequently (B (CI): 0.65 (0.45, 0.88)). As the number of people known who died from COVID-19 increased, the frequency of information seeking also increased (B (CI): 0.26 (0.14, 0.38)). Tables 1 and 2 provide information on reference groups for all demographic variables.

**Table 2:** Demographic associations with information seeking at baseline

Demographic Variables (REF = reference group)	Info-seeking B (CI), p value	Risk Perception B (CI), p value		Risk Mitigation B (CI), p value
Gender (Female)	-0.14 (-0.23, -0.05), 0.003	2.83 (0.88, 4.79), 0.004		-0.04 (-0.13, 0.06), 0.427
Age	0.01 (0.01, 0.02), < 0.001	-0.14 (-0.20, -0.07), < 0.001		0.00 (-0.00, 0.01), 0.132
Hispanic/Latinx (REF: White)	-0.09 (-0.24, 0.07), 0.276	1.78 (-1.49, 5.04), 0.286		-0.02 (-0.18, 0.13), 0.789
Asian (REF: White)	-0.02 (-0.23, 0.203), 0.896	0.99 (-3.60, 5.58), 0.673		0.29 (0.07, 0.51), 0.009
Black American (REF: White)	0.17 (0.01, 0.32), 0.035	-3.71 (-6.94, -0.47), 0.025		-0.06 (-0.21, 0.09), 0.452
Multi-racial (REF: White)	-0.03 (-0.27, 0.21), 0.811	-1.52 (-6.62, 3.59), 0.560		-0.04 (-0.29, 0.20), 0.719
Education (< HS vs. Graduate Degree)	-0.69 (-1.00, -0.38)), < 0.001	-3.86 (-10.44, 2.72), 0.250		-0.64 (-0.95, -0.34), < 0.001
Work Status (Unemployed, not looking vs. retired)	-0.65 (-0.88, -0.43), < 0.001	4.23 (-0.54, 9.00), 0.082		-0.52 (-0.74, -0.29), < 0.001
Political Party (Republican vs. Democrat)	-0.24 (-0.36, -0.13), < 0.001	-7.18 (-9.63, -4.72), < 0.001		-0.35 (-0.47, -0.24), < 0.001
#Known who have died of COVID-19	0.26 (0.14,, 0.38), < 0.001	3.91 (1.39, 6.43), 0.002		0.19 (0.08, 0.31), 0.001

Statistical significance set at 0.001. Demographic models were run for each outcome with similar results. Consequently, the same demographics variables were used for adjustment across the three outcomes models, with ethnicity and race adjusted for in each model on theoretical grounds

Next, we examined univariable models of the association between mental health at baseline and information seeking behavior, risk perception, and risk mitigation self-efficacy over time. In univariable analyses, information seeking was significantly associated with baseline distress. After adjusting for demographics (age, gender, race/ethnicity, income, number of people known who died of COVID-19, education, political party, and work status), every 1 unit increase in distress resulted in 0.06 (CI: 0.04, 0.07) units increase of information seeking frequency (p-value < 0.001). Fear, avoidance, anger, and diminished functional ability at baseline were all also associated with increased frequency of information seeking after adjusting for demographics. In general, as time went on, the frequency of information seeking decreased. The relationship between baseline mental health factors and information seeking was consistent across survey waves.

Risk perception was significantly positively associated with all baseline mental health factors (Table 3). After adjusting for demographics, every 1 unit increase in fear resulted in a 4.26% point (CI: 3.12, 5.40) increase in the perceived risk of getting COVID-19. In general, risk perception was greatest during time point 2. The relationship between mental health factors at baseline and risk perception was not dependent on time.

**Table 3:** Univariable model results for the association between baseline mental health and information seeking, risk perception, and mask wearing self-efficacy over time

	*Info-seeking* *B (CI), p value*	*Risk Perception* *B (CI), p value*	*Mask wearing self-efficacy* *B (CI), p value*
*Distress (Scale 0–10)*	*0.06 (0.04, 0.07), < 0.001*	*2.12 (1.78, 3.46), < 0.001*	*0.01 (-0.01, 0.02), 0.471*
*Wave (REF = Baseline)*			
*2*	-*0.03 (-0.14, 0.08),), 0.584*	*8.46 (6.26, 10.67)*, *< 0.001*	*0.17 (0.08, 0.27)*, *< 0.001*
*3*	*-0.09 (-0.20, 0.03), 0.145*	*8.85 (6.42, 11.29)*, *< 0.001*	*0.27 (0.16, 0.37)*, *< 0.001*
Distress * Wave (REF = Baseline)			
*2*	*0.23 (0.14, 0.33), < 0.001*	*7.00 (4.99, 9.02), < 0.001*	*-0.00 (-0.09, 0.08), 0.937*
*3*	*0.10 (-0.02, 0.23), 0.104*	*5.03 (2.45, 7.61), < 0.001*	*-0.13 (-0.24, -0.02), 0.024*
*Fear*	*0.18 (0.12, 0.23), < 0.001*	*4.26 (3.12, 5.40), < 0.001*	*0.01 (-0.04, 0.06), 0.675*
*Wave (REF = Baseline)*			
*2*	*-0.17 (-0.31, 0.04), 0.013*	*2.38 (-0.47, 5.24), 0.102*	*0.24 (0.11, 0.36)*, *< 0.001*
*3*	*-0.20 (-0.36, -0.04), 0.015*	*1.03 (-2.32, 4.38), 0.546*	*0.35 (0.21, 0.50)*, *< 0.001*
Fear * Wave			
*2*	*-0.01 (-0.08, 0.07), 0.907*	*-0.07 (-1.65, 1.52), 0.937*	*-0.06 (-0.12, 0.01), 0.107*
*3*	*-0.06 (-0.15, 0.03), 0.175*	*0.49 (-1.36, 2.34), 0.602*	*-0.10 (-0.18, -0.02), 0.011*
*Upset/avoidance*	*0.08 (0.04, 0.12), < 0.001*	*2.57 (1.70, 3.44), < 0.001*	*-0.03 (-0.07, 0.01), 0.126*
*Wave (REF = Baseline)*			
*2*	*-0.18 (-0.30, -0.05), 0.009*	*2.74 (0.01, 5.47), 0.049*	*0.18 (0.06, 0.30), 0.002*
*3*	*-0.23 (-0.38, -0.07), 0.004*	*1.49 (-1.72, 4.70), 0.364*	*0.16 (0.02, 0.30), 0.021*
Upset/avoidance * Wave (REF = Baseline)			
*2*	*0.00 (-0.06, 0.06), 0.945*	*-0.24 (-1.46, 0.99), 0.703*	*-0.02 (-0.07, 0.04), 0.508*
*3*	*-0.03 (-0.10, 0.04), 0.335*	*0.19 (-1.24, 1.62), 0.798*	*0.02 (-0.05, 0.08), 0.636*
*Anger*	*0.10 (0.05, 0.15), < 0.001*	*3.66 (2.54, 4.77), < 0.001*	*-0.08 (-0.13, -0.03), 0.001*
*Wave (REF = Baseline)*			
*2*	*-0.17 (-0.31, -0.04), 0.014*	*3.13 (0.26, 6.00), 0.032*	*0.17 (0.05, 0.29), 0.007*
*3*	*-0.16 (-0.32, 0.00), 0.047*	*1.57 (-1.81, 4.94), 0.363*	*0.21 (0.06, 0.35), 0.005*
Anger * Wave (REF = Baseline)			
*2*	*0.00 (-0.08, 0.08), 0.929*	*-0.51 (-2.08, 1.06), 0.523*	*-0.02 (-0.08, 0.05), 0.655*
*3*	*-0.08 (-0.17, 0.01), 0.077*	*0.18 (-1.66, 2.02), 0.845*	*-0.01 (-0.09, 0.07), 0.803*
*Disinterest *	*0.07 (0.03, 0.12), 0.001*	*3.50 (2.59, 4.41), < 0.001*	*-0.00 (-0.04, 0.04), 0.951*
*Wave (REF = Baseline)*			
*2*	*-0.19 (-0.34, -0.05), 0.007*	*2.77 (-0.18, 5.72), 0.065*	*0.17 (0.05, 0.30), 0.008*
*3*	*-0.23 (-0.39, -0.06), 0.008*	*3.51 (0.03, 7.00), 0.048*	*0.24 (0.09, 0.39), 0.002*
Disinterest * Wave			
*2*	*0.01 (-0.05, 0.07), 0.802*	*-0.24 (-1.50, 1.03), 0.713*	*-0.01 (-0.07, 0.04), 0.648*
*3*	*-0.03 (-0.10, 0.04), 0.372*	*-0.82 (-2.30, 0.67), 0.28*	*-0.02 (-0.09, 0.04), 0.455*
*Hopelessness*	*0.07 (0.01, 0.13), 0.016*	*3.31 (2.05, 4.58), < 0.001*	*-0.11 (-0.16, -0.05), < 0.001*
*Wave (REF = Baseline)*			
*2*	*-0.22 (-0.35, -0.09)*, *0.001*	*2.66 (-0.08, 5.40), 0.057*	*0.13 (0.01, 0.25), 0.032*
*3*	*-0.25 ( -0.41, -0.10)*, *0.001*	*0.47 (-2.81, 3.74), 0.78*	*0.18 (0.05, 0.32), 0.01*
Hopelessness * Wave (REF = Baseline)			
*2*	*0.03 (-0.06, 0.11), 0.499*	*-0.27 (-2.03, 1.49), 0.767*	*0.01 (-0.06, 0.09), 0.725*
*3*	*-0.03 (-0.13, 0.07), 0.595*	*1.10 (-1.04, 3.24), 0.315*	*0.00 (-0.09, 0.10), 0.942*
*Diminished functional ability*	*0.11 (0.06, 0.16), < 0.001*	*2.82 (1.80, 3.84), < 0.001*	*-0.00 (-0.05, 0.04), 0.947*
*Wave (REF = Baseline)*			
*2*	*-0.20 (-0.33, -0.07), 0.003*	*3.78 (1.02, 6.54), 0.007*	*0.25 (0.13, 0.36)*, *< 0.001*
*3*	*-0.23 (-0.38, -0.08), 0.004*	*1.31 (-1.92, 4.54), 0.428*	*0.29 (0.15, 0.42)*, *< 0.001*
Diminished functional ability * Wave (REF = Baseline)			
*2*	*0.01 (-0.06, 0.08), 0.749*	*-0.88 (-2.30, 0.53), 0.22*	*-0.06 (-0.12, 0.00), 0.057*
*3*	*-0.04 (-0.12, 0.04), 0.351*	*0.34 (-1.33, 2.00), 0.692*	*-0.06 (-0.13, 0.01), 0.115*

Statistical significance set at p = 0.001. All models adjusted for age, income, number of people known who died of COVID-19, education, political party, and work status

Mask wearing self-efficacy (perceived ability to wear a mask), was significantly negatively associated with baseline hopelessness (Table 3). After adjusting for demographics, every 1 unit increase in hopelessness was associated with a 0.11 point decrease in perceived ability to wear a mask (CI: -0.16, -0.05). No other baseline mental health factors were associated with this outcome. In general, perceived ability to wear a mask improved over time, perhaps related to mask related messaging, experience, availability and/or affordability. The relationship between mental health factors at baseline and this variable was not however, dependent on time.

Finally, Table 4 summarizes multivariable models examining all mental health variables that were significant in the univariable models for each outcome (e.g., for information seeking: distress, fear, anger, upset/avoidance, and diminished functional ability were put together in one model with the adjustment variables). After adjusting for demographics, only interpreting results <0.001, distress and fear were associated with increased information seeking. Increased perception of risk was also significantly associated with distress and fear in multivariable models while hopelessness was significantly associated with lower mask wearing self-efficacy.

**Table 4:** Multivariable mental health at baseline models with information seeking, risk perception and mask wearing self-efficacy over time models

*Mental Health Variables*	*Information seeking* *B (CI), p value*	*Risk Perception* *B (CI), p value*	*Mask Wearing Self-Efficacy* *B (CI), p value*
*Distress*	*0.05 (0.03, 0.06), < 0.001*	*1.73 (1.37, 2.10), < 0.001*	
*Fear*	*0.13 (0.08, 0.17), < 0.001*	*1.98 (1.05, 2.92), < 0.001*	
*Anger*	*-0.03 (-0.08, 0.01), 0.117*	*0.56 (-0.37, 1.48), 0.237*	
*Disinterest*		*1.17 (0.43, 1.90), 0.002*	
*Hopelessness*		*0.34 (-0.68, 1.36), 0.517*	*-0.11 (-0.15, -0.08), < 0.001*
*Upset/ avoidance*	*0.01 (-0.02, 0.05), 0.431*	*0.78 (0.11, 1.45), 0.023*	
*Diminished functional ability*	*0.04 (0.00, 0.08), 0.061*	*-0.43 (-1.26, 0.41), 0.319*	,

Statistical significance set at p = 0.001. All models adjusted for age, income, number of people known who died of COVID-19, education, political party, and work status. This table includes any mental health variable that was significant in Table 3 in one model. For example, for info seeking, distress, fear, anger, upset/avoidance, and diminished functional ability are in the model together with the adjustment variables

## Discussion

As a whole, our results underscore multiple ways in which mental health is intertwined with individuals’ responses to the COVID-19 pandemic. First, we find that some groups, including women, Hispanic/Latinx, Black American participants, and lower income groups reported higher rates of distress than others. Although such differences may be due to factors other than the pandemic, results are consistent with literaturesuggesting that adverse mental health outcomes associated with the pandemic are not impacting all groups equally [1, 8, 13, 70, 73–79]. Such results underscore the importance of targeting specific groups for additional outreach efforts to ensure they are receiving the necessary support.

Demographic characteristics were also associated with information seeking behavior. Information seeking was more common among older persons, those with higher education, Democrats, retirees, and those who knew people who had died of COVID-19. This is consistent with literature suggesting that there is demographic variation (e.g., gender, age, race/ethnicity) in health-related information-seeking, risk perception and risk taking/mitigation behaviors [77, 80–82]. Findings have direct implications for practice, suggesting that targeted outreach and risk communication strategies are needed for specific subgroups that may be less likely to seek information.

### Mental health and information seeking, risk perception and mask-wearing self-efficacy

Controlling for demographics, baseline mental health items were consistently associated with all key outcomes across time (information seeking, risk perception and mask-wearing self-efficacy). We focus on the most robust longitudinal relationships in the following sections, significant at <0.001, examining results relative to hypotheses informed by theoretical models and previous research.

#### Information seeking

In multivariable models, distress, fear, upset/avoidance (COVID-19 specific), and diminished functional ability were all associated with increased information seeking across time. Results support the hypothesis that distress and fear symptoms on a mental health screener will be associated with increased information seeking. These results are consistent with the *General Model of Societal Risk Information Seeking and Avoiding*, with the direction of anticipated effects consistent with previous research suggesting that information seeking is an active coping strategy for decreasing anxiety [22, 23, 28].

Also based on previous research, we predicted that COVID-specific upset/avoidance symptoms on a mental health screener would be associated with *decreased* information seeking. However, null results from the most stringent longitudinal models did not support this hypothesis. Future research should further examine relationships between avoidance of conversations or activities related to events such as COVID, and associated health information seeking.

#### Risk perception

Distress and fear were also associated with increased risk perception across time. However, other negatively-valenced responses to the mental health screener (anger, hopelessness, disinterest) did not have any significant association with risk perception. This suggests that not all negative emotions are similarly associated with increased risk (as hypothesis H3 predicts), but rather that mental health symptoms are associated with risk perception linked to specific affective states (consistent with the alternative hypothesis). Findings are consistent with other research during the pandemic indicating that fear heightens perception of risk, perhaps linked to uncertainty and a lack of control [44].

#### Mask-wearing self-efficacy

Feelings of hopelessness were associated with lower perceived mask-wearing ability across time. This was the only mental health screener item associated with this outcome. These results suggest support for the alternative hypothesis indicating that individual items associated with specific affective states are better predictors of mask wearing self-efficacy than all items associated with negatively-valenced emotions. The relationship between hopelessness and decreased self-efficacy around *mask wearing* makes sense in light of previous research indicating that depression can undermine motivation to perform certain behaviors, in part due to beliefs such as pessimism or fatalism [83]. Research indicates that self-efficacy is a precursor to mitigation behaviors [84]. In line with this and findings here, targeted messaging focused on increasing mask wearing self-efficacy may particularly benefit individuals who are feeling hopeless during an emerging public health crisis. Future research should focus on such interventions, examining suggested causal pathways. Future research should also examine the relationship between barriers to engaging in risk mitigation behaviors, such as mask wearing, and the mental health implications.

### Public health implications

This study makes important contributions to the literature, capturing longitudinal perceptions and experiences during an emerging public health crisis. Results highlight the influence of mental health, and affect-related factors on information seeking, risk perception and mask wearing self-efficacy, during a disease outbreak. These results provide support for some hypotheses derived from theoretical models, and advance understanding of the role of discrete emotions as predictors of key risk-related outcomes. Furthermore, this research illuminates individual factors that can have population level impact, underscoring the importance of multi-level public health interventions during disease outbreaks.

#### Strengths

This study emphasizes the importance of considering mental health factors when designing policies and risk communication strategies associated with public health guidance. This work also advances understanding of the role mental health can play in compliance with public health guidance. Messages about disease risk and risk-mitigation behaviors may need to be adapted for communities disproportionately experiencing mental health challenges during emerging crises. For example, messages that inspire hope and emphasize ways to increase self-efficacy may be useful for those struggling with depressive symptoms. Furthermore, practitioners may consider interventions designed to address negative thought patterns typically associated with depression, in order to encourage mask wearing and other risk mitigation behaviors.

#### Limitations

Despite the strengths of this multi-site, multi-wave panel survey, limitations of the research must be considered. The study includes data from only 6 of the U.S. states, which may not be representative of the nation as a whole. Differential attrition in panel respondents over the three survey waves may influence results and conclusions. As noted, on average those participating in only the first wave of the survey were angrier and had more challenges associated with daily living attributed to mental health symptoms (by respondents), than those who participated in follow-up surveys. Results may not fully capture those with more severe levels of mental health symptoms. Survey length limitations and attempts to reduce respondent burden during the early months of the pandemic prevented the use of full-scale mental health measures (e.g., for depression), or a more comprehensive approach to measuring outcome variables such as risk perception. Additionally, it is beyond the scope of this research to determine what amount of reported information seeking is from sources considered to be credible. Information seeking may result in exposure to misinformation. Research during the pandemic suggests however, that the most common source of information seeking was government websites, often considered to be fairly reputable compared to other sources [85].

## Conclusion

Responses to a brief mental health screener are associated with information seeking, risk perception and mask-wearing self-efficacy over time, and during the early months of a public health emergency. Mental health factors are important considerations for policy-makers, public health professionals, and mental health providers during future public health crises.

## Electronic supplementary material

Below is the link to the electronic supplementary material.

Supplementary Material 1

## Data Availability

Survey materials, de-identified data and analytic code from this study can be made available by emailing the corresponding author.

## References

[1] Czeisler M, Lane RI, Petrosky E (2020). Mental Health, Substance Use, and suicidal ideation during the COVID-19 pandemic - United States, June 24–30, 2020. MMWR Morb Mortal Wkly Rep Aug.

[2] Poll KFFC. March 2020. 2020. https://www.kff.org/coronavirus-covid-19/poll-finding/kff-coronavirus-poll-march-2020/.

[3] The Implications of COVID-19 for Mental Health and Substance Use. Kaiser Family Foundation; 2021. https://www.kff.org/coronavirus-covid-19/issue-brief/the-implications-of-covid-19-for-mental-health-and-substance-use/.

[4] Rajkumar RP (2020). COVID-19 and mental health: a review of the existing literature. Asian J Psychiatr.

[5] Torales J, O’Higgins M, Castaldelli-Maia JM, Ventriglio A (2020). The outbreak of COVID-19 coronavirus and its impact on global mental health. Int J Social Psychiatry 2020/06/01.

[6] Twenge JM, Joiner TE (2020). Mental distress among U.S. adults during the COVID-19 pandemic. J Clin Psychol Dec.

[7] Browning MHEM, Larson LR, Sharaievska I (2021). Psychological impacts from COVID-19 among university students: risk factors across seven states in the United States. PLoS ONE.

[8] Jewell JS, Farewell CV, Welton-Mitchell C, Lee-Winn A, Walls J, Leiferman JA (2020). Mental Health during the COVID-19 pandemic in the United States: Online Survey. JMIR Form Res Oct.

[9] Mortazavi SS, Assari S, Alimohamadi A, Rafiee M, Shati M, Fear (2020). Loss, social isolation, and incomplete grief due to COVID-19: a recipe for a Psychiatric Pandemic. Basic Clin Neurosci Mar-Apr.

[10] Gibbs TMLPM, Ulrick Vieux DO, Myles Solan, Paul Rosenfield MD. Mental Health Disparities Among Black Americans During the COVID-19 Pandemic. Psychiatric Times. https://www.psychiatrictimes.com/view/mental-health-disparities-among-black-americans-during-covid-19-pandemic.

[11] Holingue C, Kalb LG, Riehm KE et al. Mental Distress in the United States at the beginning of the COVID-19 pandemic. Am J Public Health. 2020/11/01 2020;110(11):1628–34. 10.2105/AJPH.2020.305857. 10.2105/AJPH.2020.305857 PMC7542294 32941066

[12] Thibaut F, van Wijngaarden-Cremers PJM. Women’s Mental Health in the Time of Covid-19 Pandemic. Mini Rev Front Global Women’s Health 2020-December-08. 2020;1. 10.3389/fgwh.2020.588372. 10.3389/fgwh.2020.588372 PMC8593968 34816164

[13] Saltzman LAEL, Veronica Henry TC, Hansel. Bordnick. COVID-19 Mental Health Disparities. Health Secur. 2021;19(S1):–5. 10.1089/hs.2021.0017. 10.1089/hs.2021.0017 34014118

[14] Kessler RC, Ruhm CJ, Puac-Polanco V, Hwang IH, Lee S, Petukhova MV, Sampson NA, Ziobrowski HN, Zaslavsky AM, Zubizarreta JR. Estimated Prevalence of and Factors Associated With Clinically Significant Anxiety and Depression Among US Adults During the First Year of the COVID-19 Pandemic. JAMA Netw Open. 2022 Jun 1;5(6):e2217223. https://pesquisa.bvsalud.org/global-literature-on-novel-coronavirus-2019-ncov/resource/pt/covidwho-1888479. 10.1001/jamanetworkopen.2022.17223 PMC9201669 35704316

[15] Sun, Y., Wu, Y., Fan, S., Dal Santo, T., Li, L., Jiang, X., ... & Thombs, B. D. (2023). Comparison of mental health symptoms before and during the covid-19 pandemic: evidence from a systematic review and meta-analysis of 134 cohorts. bmj, 380 10.1136/bmj-2022-074224 PMC9992728 36889797

[16] Schäfer, M., Stark, B., Werner, A. M., Tibubos, A. N., Reichel, J. L., Pfirrmann, D., ... & Dietz, P. (2021). Health information seeking among university students before and during the corona crisis—findings from Germany. Frontiers in public health, 8, 616603 10.3389/fpubh.2020.616603 PMC7873736 33585388

[17] Anker AE, Reinhart AM, Feeley TH (2011). Health information seeking: a review of measures and methods. Patient Educ Couns.

[18] Ikoja-Odongo R, Mostert J. Information seeking behaviour: a conceptual framework. South Afr J Libr Inform Sci 01/01. 2006;7210.7553/72-3-1112.

[19] Ferrer RA, Klein WMP, Lerner JS, Reyna VF. Dacher. Emotions and Health Decision-Making: Extending the Appraisal Tendency Framework to Improve Health and Healthcare. 2014.

[20] Jacobs J, Taylor M, Agho K, Stevens G, Barr M, Raphael B (2010). Factors associated with increased risk perception of pandemic influenza in australia. Influenza Res Treat.

[21] Honda K, Goodwin RD, Neugut AI (2005). The associations between psychological distress and cancer prevention practices. Cancer Detect Prev.

[22] Lagoe C, Atkin D. Health anxiety in the digital age: An exploration of psychological determinants of online health information seeking. Computers in Human Behavior. 2015/11/01/ 2015;52:484–491. 10.1016/j.chb.2015.06.003.

[23] Myrick JG, Willoughby JF (2019). Educated but anxious: how emotional states and education levels combine to influence online health information seeking. Health Inf J Sep.

[24] Oh YS, Song NK. Investigating Relationships Between Health-Related Problems and Online Health Information Seeking. CIN:, Computers. Informatics, Nursing. 2017;35(1). 10.1097/CIN.0000000000000234 26950091

[25] Powell J, Clarke A (2006). Internet information-seeking in mental health: Population survey. Br J Psychiatry.

[26] Singh K, Brown RJ, Anxiety (2014). Stress & Coping.

[27] McMullan RD, Berle D, Arnáez S, Starcevic V (2019). The relationships between health anxiety, online health information seeking, and cyberchondria: systematic review and meta-analysis. J Affect Disord.

[28] Kahlor L. PRISM: A Planned Risk Information Seeking Model. Health Communication. 2010/05/28 2010;25(4):345–356. 10.1080/10410231003775172. 10.1080/10410231003775172 20512716

[29] Neuberger L, Silk KJ (2016). Uncertainty and information-seeking patterns: a test of competing hypotheses in the context of health care reform. Health Commun.

[30] Baumgartner SE, Hartmann T, Social Networking. The role of health anxiety in Online Health Information Search. Cyberpsychology, Behavior, and. 2011/10/01 2011;14(10):613–8. 10.1089/cyber.2010.0425. 10.1089/cyber.2010.0425 21548797

[31] Barbour JB, Rintamaki LS, Ramsey JA, Brashers DE (2012). Avoiding Health Information. J Health Communication 2012/02/01.

[32] Holmes BJ (2008). Communicating about emerging infectious disease: the importance of research. Health Risk & Society.

[33] Thompson RA. Regulation: a theme in, search of definition. Monogr Soc Res Child Dev. 1994;59(2–3):25–52. 10.1111/j.1540-5834.1994.tb01276.x. 7984164

[34] Holahan CJ, Moos RH, Bonin LA. Social context and depression: an integrative stress and coping framework. The interactional nature of depression: advances in interpersonal approaches. American Psychological Association; 1999. pp. 39–63.

[35] Stress Generation A, Coping, Symptoms D. A 10-Year model. American Psychological Association; 2005. 10.1037/0022-006X.73.4.658.

[36] Novotney A. The risks of social isolation. Monitor on Psychology. https://www.apa.org/monitor/2019/05/ce-corner-isolation.

[37] Ottenbreit ND, Dobson KS, Quigley L. An examination of avoidance in major depression in comparison to social anxiety disorder. Behaviour Research and Therapy. 2014/05/01/ 2014;56:82–90. 10.1016/j.brat.2014.03.005. 10.1016/j.brat.2014.03.005 24727363

[38] Barrett EL, Mills KL, Teesson M (2013). Mental health correlates of anger in the general population: findings from the 2007 National Survey of Mental Health and Wellbeing. Australian & New Zealand Journal of Psychiatry 2013/05/01.

[39] Myrick JG, Willoughby JF. Educated but anxious: How emotional states and education levels combine to influence online health information seeking. Health Informatics Journal., Ferrer R, Klein WM. Risk perceptions and health behavior. Curr Opin Psychol. 2015 Oct 1;5:85–89. 10.1016/j.copsyc.2015.03.012. PMID: 26258160; PMCID: PMC4525709.

[41] Tagini S, Brugnera A, Ferrucci R (2021). It won’t happen to me! Psychosocial factors influencing risk perception for respiratory infectious diseases: a scoping review. Appl Psychology: Health Well-Being.

[42] Koh D, Lim MK, Chia SE (2005). Risk perception and impact of severe Acute Respiratory Syndrome (SARS) on work and personal lives of healthcare workers in Singapore: what can we learn?. Med Care Jul.

[43] Rovner BW, Haller JA, Casten RJ, Murchison AP, Hark LA (2014). Depression and risk perceptions in older African Americans with Diabetes. Diabetes Spectr.

[44] Lanciano T, Graziano G, Curci A, Costadura S, Monaco A. Risk Perceptions and Psychological Effects During the Italian COVID-19 Emergency. Original Research. Frontiers in Psychology. 2020-September-18 2020;11doi:10.3389/fpsyg.2020.58005345. Yang, C. (2020). Does hand hygiene reduce SARS-CoV-2 transmission?. Graefe’s Archive for Clinical and Experimental Ophthalmology, 258, 1133–1134.

[46] Li ASW, Figg G, Schüz B. Socioeconomic status and the prediction of health promoting dietary behaviours: a systematic review and meta-analysis based on the theory of planned behaviour. Appl Psychol Health Well-Being. 2019;11(3):382–406. 10.1111/aphw.12154. 10.1111/aphw.12154 30884154

[47] McEachan RRC, Conner M, Taylor NJ, Lawton RJ (2011). Prospective prediction of health-related behaviours with the theory of Planned Behaviour: a meta-analysis. Health Psychol Rev 2011/09/01.

[48] Morley KI, Lynskey MT, Moran P, Borschmann R, Winstock AR. Polysubstance use, mental health and high-risk behaviours: Results from the 2012 Global Drug Survey. Drug Alcohol Rev. 2015;34(4):427–37. 10.1111/dar.12263. 10.1111/dar.12263 25867685

[49] Harper CA, Satchell LP, Fido D, Latzman RD (2021). Functional fear predicts Public Health Compliance in the COVID-19 pandemic. Int J Ment Health Addict.

[50] Ning L, Niu J, Bi X et al. The impacts of knowledge, risk perception, emotion and information on citizens’ protective behaviors during the outbreak of COVID-19: a cross-sectional study in China. BMC Public Health. 2020/11/23 2020;20(1):1751. 10.1186/s12889-020-09892-y. 10.1186/s12889-020-09892-y PMC7681179 33225934

[51] Mertens G, Gerritsen L, Duijndam S, Salemink E, Engelhard IM. Fear of the coronavirus (COVID-19): predictors in an online study conducted in March 2020. J Anxiety Disord. 2020/08/01/ 2020;74:102258. 10.1016/j.janxdis.2020.102258. 10.1016/j.janxdis.2020.102258 PMC7286280 32569905

[52] Ludman EJ, Ichikawa LE, Simon GE et al. Breast and Cervical Cancer Screening: Specific Effects of Depression and Obesity. American Journal of Preventive Medicine. 2010/03/01/ 2010;38(3):303–310. 10.1016/j.amepre.2009.10.039. 10.1016/j.amepre.2009.10.039 PMC2835516 20171532

[53] Kaida A, Colman I, Janssen PA (2008). Recent pap tests among canadian women: is Depression a barrier to Cervical Cancer Screening?. J Women’s Health 2008/09/01.

[54] Bodas M, Siman-Tov M, Kreitler S, Peleg K (2017). Psychological correlates of civilian preparedness for conflicts. Disaster Med Pub Health Prep.

[55] James LE, Welton-Mitchell C, Noel JR, James AS (2020). Integrating mental health and disaster preparedness in intervention: a randomized controlled trial with earthquake and flood-affected communities in Haiti. Psychol Med Jan.

[56] Karimy M, Koohestani HR, Araban M. The association between attitude, self-efficacy, and social support and adherence to diabetes self-care behavior. Diabetol Metab Syndr. 2018/11/27 2018;10(1):86. 10.1186/s13098-018-0386-6. 10.1186/s13098-018-0386-6 PMC6260748 30534204

[57] Clark C, Davila A, Regis M, Kraus S (2020). Predictors of COVID-19 voluntary compliance behaviors: an international investigation. Glob Transit.

[58] Koebele EA, Albright EA, Dickinson KL, Perceptions of Efficacy are Key Determinants of Mask-Wearing Behavior during the COVID-19 Pandemic. Nat Hazards Rev. 2021;22(3):06021002. 10.1061/(ASCE)NH.1527-6996.0000489.

[59] Burger R, Christian C, English R, Maughan-Brown B, Rossouw L et al. Predictors of mask-wearing during the advent of the COVID-19 pandemic: evidence from South Africa. Transl Behav Med. 2022;12(1):ibab132. 10.1093/tbm/ibab132. 10.1093/tbm/ibab132 PMC8690224 34865174

[60] Shacham M, Hamama-Raz Y, Kolerman R, Mijiritsky O, Ben-Ezra M, Mijiritsky E. COVID-19 factors and psychological factors Associated with elevated psychological distress among dentists and Dental Hygienists in Israel. Int J Environ Res Public Health Apr. 2020;22(8). 10.3390/ijerph17082900. 10.3390/ijerph17082900 PMC7215275 32331401

[61] Yıldırım M, Güler A (2022). Factor analysis of the COVID-19 perceived risk scale: a preliminary study. Death Stud 04/17.

[62] Gross JJ, Uusberg H, Uusberg A (2019). Mental illness and well-being: an affect regulation perspective. World Psychiatry.

[63] Choo CW. Seeking and avoiding information in a risky world. Inf Res. 2017;22.

[64] Godovykh M, Pizam A, Bahja F. Antecedents and outcomes of health risk perceptions in tourism, following the COVID-19 pandemic. Tourism Rev 01/27 2021;ahead-of-printdoi:10.1108/TR-06-2020-0257.

[65] Lerner JS, Han S, Keltner D. Feelings and Consumer Decision Making: Extending the Appraisal-Tendency Framework. Journal of Consumer Psychology. 2007/07/01/ 2007;17(3):181–187. 10.1016/S1057-7408(07)70027-X.

[66] Lerner JS, Keltner D (2000). Beyond valence: toward a model of emotion-specific influences on judgement and choice. Cogn Emot.

[67] Ownby KK (2019). Use of the Distress Thermometer in Clinical Practice. J Adv Pract Oncol.

[68] World Health Organization U. Assessing mental health and psychosocial needs and resources: toolkit for humanitarian settings. World health Organization; 2012.

[69] Roberts JD, Dickinson KL, Koebele E (2020). Clinicians, cooks, and cashiers: examining health equity and the COVID-19 risks to essential workers. Toxicol Ind Health Sep.

[70] Census Bureau US, Reports CP. P60-276, income in the United States: 2021. Washington, DC: U.S. Government Publishing Office; September 2022.

[71] Ownby KK. Use of the Distress Thermometer in Clinical Practice. J Adv Pract Oncol. 2019 Mar;10(2):175–9. Epub 2019 Mar 1. PMID: 31538028; PMCID: PMC6750919. PMC6750919 31538028

[72] World Health Organization. Assessing mental health and psychosocial needs and resources: Toolkit for humanitarian settings. World Health Organization; 2012.

[73] Kluger J. The Coronavirus Pandemic’s Outsized Effect on Women’s Mental Health Around the World. The New York Times. https://time.com/5892297/women-coronavirus-mental-health/.

[74] Liu X, Zhu M, Zhang R et al. Public mental health problems during COVID-19 pandemic: a large-scale meta-analysis of the evidence. Translational Psychiatry. 2021/07/09 2021;11(1):384. 10.1038/s41398-021-01501-9. 10.1038/s41398-021-01501-9 PMC8266633 34244469

[75] McKnight-Eily LR, Okoro CA, Strine TW et al. Racial and Ethnic Disparities in the Prevalence of Stress and Worry, Mental Health Conditions, and Increased Substance Use Among Adults During the COVID-19 Pandemic - United States, April and May 2020. MMWR Morbidity and mortality weekly report. 2021 2021;70(5):162–166. 10.15585/mmwr.mm7005a3. 10.15585/mmwr.mm7005a3 PMC7861483 33539336

[76] Raifman J, Bor J, Venkataramani A (2021). Association between receipt of unemployment insurance and food insecurity among people who lost employment during the COVID-19 pandemic in the United States. JAMA Netw Open.

[77] Smith K, Bhui K, Cipriani A (2020). COVID-19, mental health and ethnic minorities. Evid Based Mental Health.

[78] Taquet M, Luciano S, Geddes JR, Harrison PJ. Bidirectional associations between COVID-19 and psychiatric disorder: retrospective cohort studies of 62 354 COVID-19 cases in the USA. The Lancet Psychiatry. 2021;8(2):130–140. 10.1016/S2215-0366(20)30462-4 10.1016/S2215-0366(20)30462-4 PMC7820108 33181098

[79] Wilson JM, Lee J, Fitzgerald HN, Oosterhoff B, Sevi B, Shook NJ. Job insecurity and financial concern during the COVID-19 pandemic are Associated with worse Mental Health. J Occup Environ Med. 2020;62(9). 10.1097/JOM.0000000000001962 32890205

[80] Fareed N, Swoboda CM, Jonnalagadda P, Walker DM, Huerta TR (2020). Differences between races in Health Information seeking and Trust over Time: evidence from a cross-sectional, pooled analyses of HINTS Data. Am J Health Promotion 2021/01/01.

[81] Finucane ML, Slovic P, Mertz CK, Flynn J, Satterfield TA. Gender, race, and perceived risk: The ‘white male’ effect. Health Risk & Society. 2000/07/01 2000;2(2):159–72. 10.1080/713670162.

[82] Rhodes N, Pivik K (2011). Age and gender differences in risky driving: the roles of positive affect and risk perception. Accid Anal Prev May.

[83] Katayama N, Nakagawa A, Umeda S et al. Frontopolar cortex activation associated with pessimistic future-thinking in adults with major depressive disorder. NeuroImage: Clin. 2019/01/01/ 2019;23:101877. 10.1016/j.nicl.2019.101877. 10.1016/j.nicl.2019.101877 PMC6551553 31170685

[84] Green EC, Murphy EM, Gryboski K. The Health Belief Model. The Wiley Encyclopedia of Health Psychology. 2020:211–214.

[85] Ali SH, Foreman J, Tozan Y, Capasso A, Jones AM, DiClemente RJ (2020). Trends and Predictors of COVID-19 information sources and their relationship with knowledge and beliefs related to the pandemic: Nationwide Cross-Sectional Study. JMIR Public Health Surveill.

