# Supplementary material for: Influence of mental health on information seeking, risk perception and mask wearing self-efficacy during the early months of the COVID-19 pandemic: a longitudinal panel study across 6 U.S. States
Source: BMC Psychol. 2023 Jul 10;11:203. doi: 10.1186/s40359-023-01241-z (PMC10334593; doi:10.1186/s40359-023-01241-z)
Supplement: Supplementary file 1 — Supplementary Material 1 [file 40359_2023_1241_MOESM1_ESM.docx]

**Supplemental Material**

Annex I. Comparison between those included in the longitudinal cohort and those lost to follow up after the first survey. Statistical significance set at p=0.001.

|  | ***Participated in Baseline Only***  ***(N = 827)*** | ***Longitudinal Cohort***  ***(N = 2232)*** | ***p-value*** |
| --- | --- | --- | --- |
| **Primary outcomes** |  |  |  |
| Risk perception - Chance of getting COVID | 27.14 (23.62) | 27.99 (22.75) | 0.369 |
| Risk perception - Chance of serious illness from COVID | 33.17 (26.77) | 34.35 (27.23) | 0.281 |
| Risk perception - Chance of dying from COVID | 21.59 (25.70) | 21.82 (25.34) | 0.828 |
| How closely been following news and info | 2.13 (1.11) | 2.24 (1.08) | 0.016 |
| Able to wear a face mask | 4.32 (0.99) | 2.89 (1.07) | 0.394 |
| **Primary predictors** |  |  |  |
| Distress (Scale 1-10) | 4.62 (2.87) | 4.62 (2.79) | 0.99 |
| Distress (Yes; > 5) | 344 (42%) | 930 (42%) | 0.972 |
| Fear | 1.70 (0.97) | 1.59 (0.87) | 0.003 |
| Anger | 1.70 (0.97) | 1.61 (0.88) | 0.001 |
| Disinterest | 2.11 (1.11) | 2.07 (1.08) | 0.325 |
| Hopelessness | 1.46 (0.92) | 1.36 (0.80) | 0.008 |
| Upset/Avoidant | 2.00 (1.18) | 1.92 (1.12) | 0.117 |
| Diminished functional ability | 1.90 (1.15) | 1.70 (0.98) | <0.001 |
| **Characteristics** |  |  |  |
| Household size | 3.27 (2.05) | 3.08 (1.64) | 0.017 |
| Children | 0.80 (1.11) | 0.81 (1.12) | 0.969 |
| Age | 42.17 (17.21) | 43.28 (15.58) | 0.105 |
| Number of people known COVID | 4.55 (19.28) | 3.81 (17.40) | 0.333 |
| Number of people known hospitalized | 3.64 (17.87) | 3.56 (17.68) | 0.909 |
| Number of people known died | 0.18 (0.38) | 0.17 (0.38) | 0.946 |
| Chance of running out of money due to COVID | 29.73 (33.13) | 26.96 (31.87) | 0.039 |
| **State** |  |  | <0.001 |
| Colorado | 146 (18%) | 366 (16%) |  |
| Iowa | 157 (19%) | 350 (16%) |  |
| Louisiana | 154 (19%) | 358 (16%) |  |
| Massachusetts | 130 (16%) | 381 (17%) |  |
| Michigan | 100 (12%) | 418 (19%) |  |
| Washington | 140 (17%) | 359 (16%) |  |
| **Gender** |  |  |  |
| Female | 435 (53%) | 1403 (63%) | <0.001 |
| **Race/Ethnicity** |  |  | <0.001 |
| Hispanic/Latinx | 112 (14%) | 211 (10%) |  |
| Non-Hispanic/Latinx - Asian | 21 (3%) | 100 (5%) |  |
| Non-Hispanic/Latinx - Black American | 116 (14%) | 215 (10%) |  |
| Non-Hispanic/Latinx - Other or Multiracial | 38 (5%) | 80 (4%) |  |
| Non-Hispanic/Latinx - White | 540 (65%) | 1626 (73%) |  |
| **Income** |  |  | 0.012 |
| $10,000 or less | 90 (11%) | 181 (8%) |  |
| $10,001-$20,000 | 83 (10%) | 172 (8%) |  |
| $20,001-$30,000 | 65 (8%) | 180 (8%) |  |
| $30,001 to $40,000 | 65 (8%) | 172 (8%) |  |
| $40,001 to $50,000 | 58 (7%) | 192 (9%) |  |
| $50,001 to $60,000 | 62 (8%) | 167 (8%) |  |
| $60,001 to $80,000 | 94 (11%) | 269 (12%) |  |
| $80,001 to $100,000 | 66 (8%) | 266 (12%) |  |
| $100,001 to $150,000 | 161 (20%) | 289 (17%) |  |
| More than $150,000 | 83 (10%) | 241 (11%) |  |
| **Education** |  |  | 0.028 |
| Less than high school | 32 (4%) | 52 (2%) |  |
| High school | 177 (21%) | 418 (19%) |  |
| Some college | 193 (23%) | 535 (24%) |  |
| 2 year degree | 82 (10%) | 264 (12%) |  |
| 4 year degree | 192 (23%) | 588 (26%) |  |
| Graduate degree | 151 (18%) | 375 (17%) |  |
| **Political Party** |  |  | 0.282 |
| Democrat | 306 (37%) | 797 (36%) |  |
| Republican | 178 (22%) | 551 (25%) |  |
| Independent | 288 (35%) | 756 (34%) |  |
| Other | 55 (7%) | 128 (6%) |  |
| **Employment status** |  |  | 0.035 |
| Employed full time (32 or more hours/week) | 346 (42%) | 1002 (45%) |  |
| Employed part time (1-31 hours/week) | 134 (16%) | 270 (12%) |  |
| Working without pay (e.g., taking care of children, volunteering, etc.) | 22 (3%) | 94 (4%) |  |
| Furloughed | 35 (4%) | 97 (4%) |  |
| Unemployed and looking for work | 76 (9%) | 174 (8%) |  |
| Unemployed and not looking for work | 35 (4%) | 123 (6%) |  |
| Receiving or awaiting approval for disability payments | 28 (3%) | 82 (4%) |  |
| Primarily a student | 40 (5%) | 100 (5%) |  |
| Retired | 11 (28%) | 290 (13%) |  |
| Unemployed due to COVID | 56 (54%) | 129 (45%) | 0.134 |
| Essential worker | 280 (38%) | 771 (36%) | 0.538 |
| **Had COVID** | 69 (10%) | 203 (11%) | 0.481 |
